# Continuous hypergammaglobulinemia and proteinuria after the recovery of the visceral Leishmaniasis: a case report

**DOI:** 10.1186/s12879-021-05819-z

**Published:** 2021-01-28

**Authors:** Linfeng Zou, Gang Chen, Yangzhong Zhou, Wei Ye, Yubin Wen, Limeng Chen, Xuemei Li

**Affiliations:** 1 Department of Internal Medicine, Peking Union Medical College Hospital, Peking Union Medical College, Chinese Academy of Medical Sciences, Beijing, 100730 China; 2 Department of Nephrology, Peking Union Medical College Hospital, Peking Union Medical College, Chinese Academy of Medical Sciences, Beijing, 100730 China

**Keywords:** Case report, Visceral Leishmaniasis, Hypergammaglobulinemia

## Abstract

**Background:**

Kidney involvement of visceral Leishmaniasis is previously reported, but knowledge is limited. Hypergammaglobulinemia is common in visceral leishmaniasis patients. Whether hypergammaglobulinemia after leishmaniasis depletion can cause kidney injury is not well reported yet.

**Case presentation:**

We reported a patient who recovered from visceral Leishmaniasis but showed persistent hypergammaglobulinemia and elevated urinary protein. Kidney biopsy showed glomerular hypertrophy with mild segmental mesangial proliferation without tubulointerstitial involvement in light microscopy. No immune complex deposit was found in the mesangial area by neither immunofluorescent staining nor electronic microscope. Increased lysosomes were observed in proximal tubules by electronic microscope. Valsartan was administered to decrease urinary protein, and no immune-suppressive therapy was added. The urinary protein and serum IgG level gradually dropped, and serum creatinine level remained stable during three- month follow up.

**Conclusions:**

Hypergammaglobulinemia is unlikely to cause renal structural or functional damage in the short term. Angiotensin blockade significantly reduced urine protein, with a minor effect on IgG elimination.

## Case presentation

A 30-year-old male visited our outpatient clinic in March 2019, presented with irregular fever and newly-onset proteinuria. He was from Shanxi province in the northwestern part of China. His fever started in April 2018, with the highest body temperature reaching 40 °C. Meanwhile, he only experienced a slight cough. Doctors in local hospitals found an enlarged spleen by physical examination, while the routine tests were mostly normal, including complete blood count (CBC), urinalysis, and tests for liver functions. His serum creatinine (SCr) was 72.5 μmol/L, and total serum protein level was 102.65 g/L, dominated with immunoglobulin (Ig) G (51.2 g/L). The inflammatory markers were elevated (C reactive protein 31.93 mg/L, erythrocyte sedimentation rate 68 mm/h). Abdominal ultrasound revealed enlarged spleen and kidneys. Empirical antibiotic and antiviral treatments were administered without significant effect. The patient reported a weight loss of 25 kg within 3 months. In October 2018, a positron emission tomography-computed tomography scanning showed an enlarged spleen with a longitudinal diameter of 30 cm and a standardized uptake value (SUV) of 5.92. The max SUV of his bone marrow also reached 4.66. Malignant diseases were suspected, and he underwent bone marrow biopsy and splenectomy. Pathology of bone marrow indicated scattered infiltration of the plasma cell, and necrosis with reactive hyperplasia was seen in the spleen. Further tests for fusion genes, chromosomes, and fluorescence in situ hybridization (FISH) were not remarkable. His fever and increased serum IgG remained after the splenectomy, while SCr increased to 100 μmol/L. Repeated urinalysis indicated moderate protein and blood. His past medical history was generally nonremarkable.

**Fig. 1:**
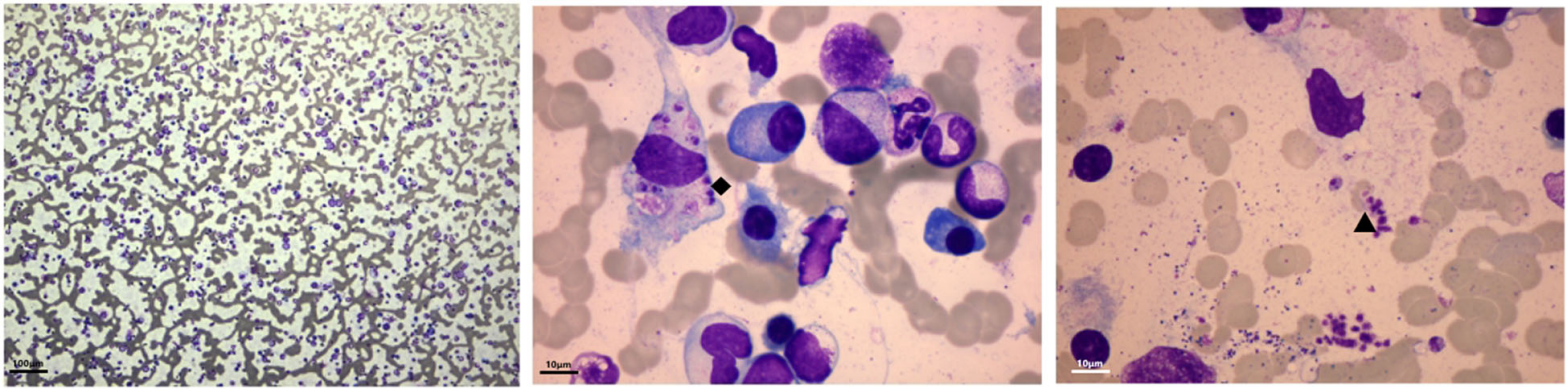
Amastigote forms of Leishmania was visible inside (◆) and outside (▲) of phagocytes

In March 2019, his tests in our outpatient clinic showed proteinuria (24-h urine protein 6.88 g) and hypoalbuminemia (serum albumin 23 g/L). His SCr was 93 μmol/L, and serum IgG level was higher than the normal upper range (99 g/L), dominated with IgG1 (106 g/L). None monoclonal protein was found after performing serum protein electrophoresis, immunofixation electrophoresis, and urine free light chain test. Finally, we repeated bone marrow biopsy and found Leishmania inside and outside the phagocytes (Fig. 1). A diagnosis of Leishmaniasis was established, but inquiries of travel history or contact history were nonremarkable. From March 2019 to October 2019, the patient underwent treatments of sodium antimony gluconate (Table 1), after which a repeated bone marrow biopsy proved the clearance of Leishmania. However, the elevation of his serum IgG level and urine protein persisted.

**Table 1:** Treatment of leishmaniasis and monitoring of renal functions

Round	Regimen (sodium antimony gluconate)	Albumin (g/L)	Creatine (μmol/L)	Serum IgG (g/L)	24hUP (g)
1st	600 mg/day im × 2 days →300 mg/day im × 7 days	20.6	69.4	125.5	6.88
2nd	600 mg/day im × 9 days	26.3	71.5	113	6.36
3rd	600 mg/day im × 6 days	27.7	55.0	62.3	3.98

*Abbreviation*: *Ig* immunoglobulin, *24hUP* 24-h urine protein; im: intramuscular injection, *mg* milligram

**Fig. 2:**
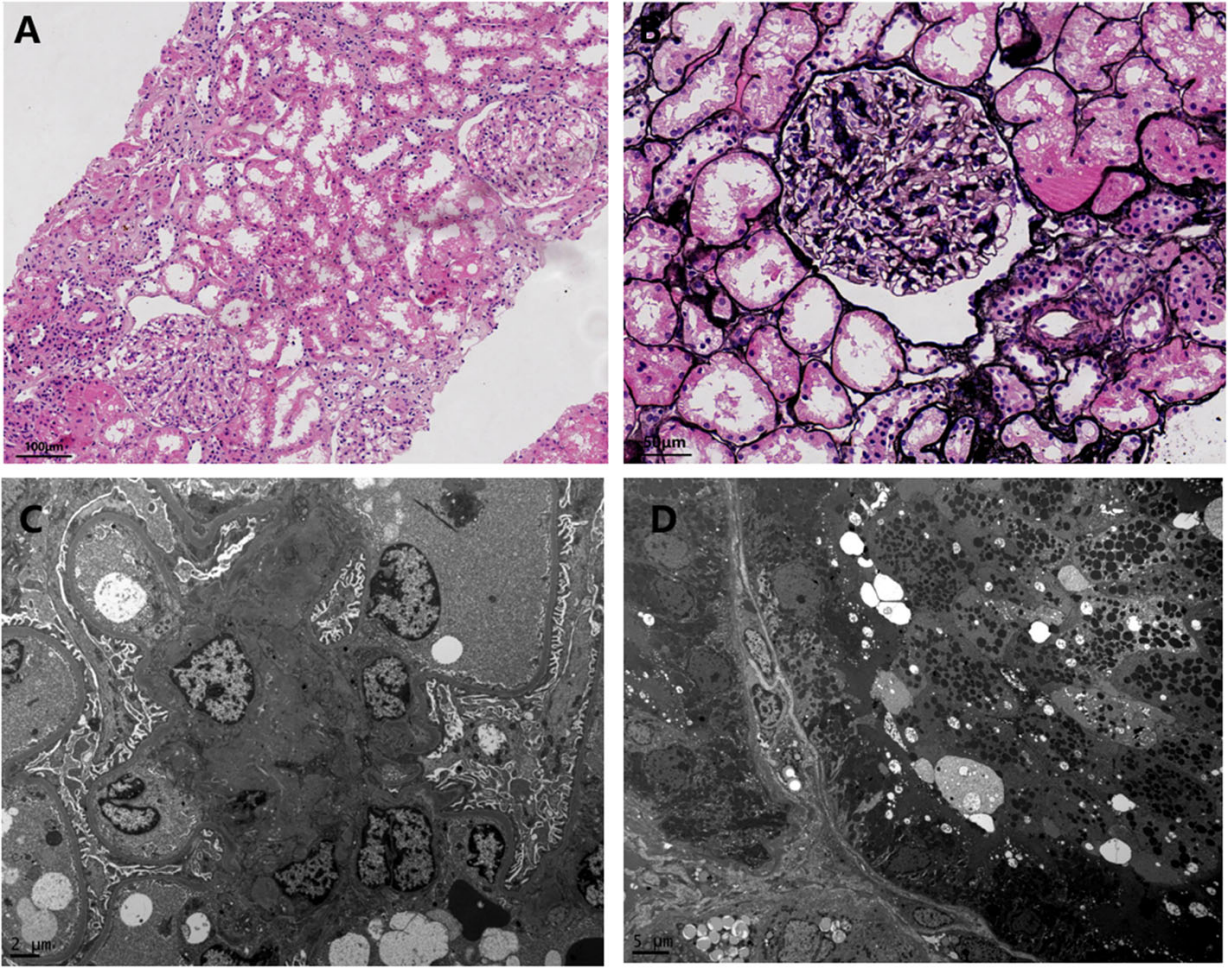
In the light microscope, glomerulus was hypertrophy (**a**, HE staining), and segmental mesangial proliferation(**b**, PASM staining) could be observed. **b**. In the electronic microscope, few electronic density deposited in the mesangial area (**c**), but increased lysosomes could be observed in proximal tubules (**d**). Note: The immunofluorescent results were all negative for IgG, IgA, IgM, C3, C4, C1q

In November 2019, he was admitted to our nephrology department. Physical examination was mostly normal, without palpable lymph nodes or extremity edema. His SCr and proteinuria improved (SCr 58 μmol/L, 24-h urine protein 3.71 g, serum albumin 33 g/L). His urine protein was composed of albumin (45.6%), IgG (39.6%), transferrin (9.8%), free light chain (1.0%), and unknown proteins (4.0%), as shown by the electrophoresis. The flow cytometry of peripheral blood mononuclear cells revealed elevated count B cells (894/μL) and normal counts of T cells and natural killer cells. The urinary transferrin, β2-MG, and α1-MG were also above the normal upper range. The longitudinal diameters of kidneys, measured by ultrasound, were l5.6cm and 14.7 cm, respectively. A renal biopsy was performed. Light microscopy showed mild segmental mesangial proliferation and glomerular hypertrophy, without tubulointerstitial lesions (Fig. 2a, b). The immunofluorescent stainings of immunoglobulin and complements were negative. Electron microscope showed the increased lysosomes in proximal tubules, but immune complex deposits were not found in the mesangial area (Fig. 2c, d).

**Fig. 3:**
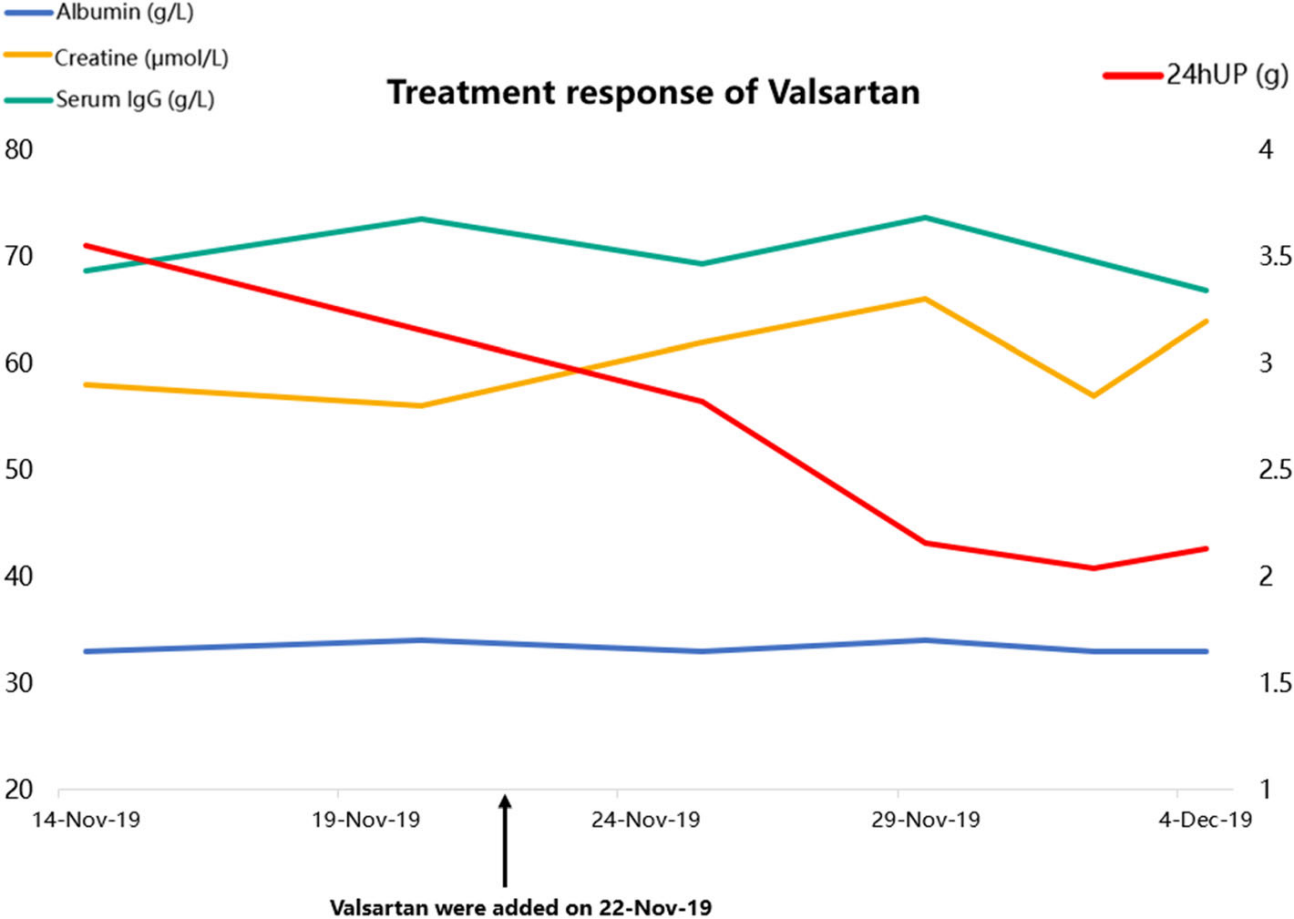
Treatment response of Valsartan. During hospitalization, the albumin, serum creatinine, and IgG level remained stable, while 24hUP dropped significantly after the addition of Valsartan. Three months after discharge, the IgG level dropped to 45 g/L, and the 24hUP level dropped to 1.59 g

Based on renal biopsy findings, we did not recommend immune-suppressive therapies but administrated Valsartan to decrease urinary protein. The urine protein gradually dropped to 1.59 g during the following 3 months, and the serum IgG level decreased to 45 g/L. His serum albumin and SCr levels remained stable (Fig. 3).

## Discussion

Leishmaniasis consists of a complex of vector-borne diseases caused by the protozoa genus *Leishmania.* The L. *infantum* and *L. donovani* are common types in west China [1]. Visceral Leishmaniasis leads to multi-organ involvements. A recent case series suggested hepatosplenomegaly was one of the most common symptoms in children [2]. But other organs, like the kidney, could also be involved. However, understandings of kidney involvement are still limited. Clinical observation revealed acute kidney injury [3] and renal tubular dysfunction [4]. Pathologically, both glomerulonephritis [5] and interstitial nephritis [6] have been reported. Glomerular involvement included mesangial proliferative glomerulonephritis [7], focal segmental glomerulosclerosis [8], and membranoproliferative glomerulonephritis [9]. Other kidney impairment might attribute to co-morbidities, such as viral infection [10] or side effects of drugs [11].

The clinical manifestations were featured with polyclonal hypergammaglobulinemia in this patient. The peak of his serum IgG level was above 100 g/L, mainly consisting of IgG1. Similar findings were observed in patients with visceral leishmaniasis [4, 12]. A mice experiment suggested that the extremely high IgG might be caused by endosomal Toll-like receptor activation [13]. It was previously assumed that the immune complex deposits lead to kidney injury in the hamster model [14]. However, whether the hypergammaglobulinemia has a pathogenic role in human kidney injury remained unknown. In this patient, the negative immunofluorescence and the lack of immune deposits in the electronic microscope suggested the serum IgG might not get involved in pathogenesis. Thus, we did not employ aggressive approaches, such as plasma exchange or immunosuppressive therapies, to eliminate IgG or reduce IgG production. During the follow-up, the patient’s renal function remained stable, and serum IgG dropped gradually. This further proved the self-limiting feature of these abnormalities after the eradication of Leishmaniasis.

Angiotensin blockage can reduce proteinuria by reducing intraglomerular pressure [15]. A considerable amount of the urinary protein in this patient was IgG, which arose the concern that lowering urinary protein might simultaneously lower IgG clearance, extending the disease course. Either excretion or catabolism physiologically eliminates antibodies. The kidney is not the primary way to eliminate antibodies [16, 17]. In normal conditions, IgG is too large to be filtered through glomeruli. If low molecular weight antibody fragments are filtered, they are usually reabsorbed and metabolized in the proximal tubules [18]. The increased urinary IgG level in our patient suggests some unknown deficiency in glomerular filtration barriers, but whether this is related to Leishmaniasis cannot be proven by renal biopsy. From a clinical perspective, lowering IgG elimination by urine might only have a minor effect on overall IgG clearance, and high IgG filtration burden does not influence glomerular structure and function in the short term. As indicated in this case, the urine protein and serum IgG levels simultaneously reduced after Valsartan administration.

Some tubular injury markers, like β2- and α1- microglobulins, is elevated in the urine. In the electronic microscope, increased lysosomes are detected. This might suggest that proximal tubular injury happens without clinical significance. A case series of visceral Leishmaniasis reported clinical presentations of proximal tubular damage with alteration in low-molecule protein, glucose, and uric acid reabsorption [4]. Another leishmaniasis case series reported elevated kidney injury molecule-1, a proximal tubule injury biomarker [3]. The apical exposure to protein overload in proximal tubular cells might induce a proinflammatory phenotype, leading to injury [19]. Our patient has a large amount of IgG secretion, which might trigger proximal tubular cell reabsorption and induce cell damage. However, we do not find electrolyte abnormal in our patients, nor observe tubulointerstitial lesions in the light microscope. It seems that some tubular dysfunction in visceral Leishmaniasis is reversible after pentavalent antimony treatment, but some may persist [20]. Whether the damage in proximal tubular cells has clinical significance or impact on prognosis in our patient requires a longer follow-up.

In conclusion, we present a patient with significant hypergammaglobulinemia and proteinuria after recovery from visceral Leishmaniasis by antimony treatment. The kidney pathology shows mesangial proliferative glomerulonephritis in the light microscope, but no immune complex deposits are evidenced in immunofluorescence and electronic microscope. Increased lysosomes are observed in proximal tubules without known clinical significance. Hypergammaglobulinemia is unlikely to cause short-term renal structural or functional damage. Angiotensin blockade significantly reduces urine protein, with a minor effect on IgG elimination. The renal function is stable during our follow-up. A Longer-term follow-up is still required.

## Data Availability

We presented All necessary data as tables and figures in the manuscript. Related information is accessible under request to the corresponding author.

## Abbreviations

24hUP: 24-h urine protein
Nov: November
Dec: December
